# Madelung Formalism for Electron Spill-Out in Nonlocal Nanoplasmonics

**DOI:** 10.1021/acs.jpcc.2c04828

**Published:** 2022-08-19

**Authors:** Rúben A. Alves, Víctor Pacheco-Peña, Miguel Navarro-Cía

**Affiliations:** † School of Physics and Astronomy, University of Birmingham, Birmingham B15 2TT, United Kingdom; ‡ School of Mathematics, Statistics and Physics, Newcastle University, Newcastle Upon Tyne NE1 7RU, United Kingdom; § Department of Electronic, Electrical and Systems Engineering, University of Birmingham, Birmingham B15 2TT, United Kingdom

## Abstract

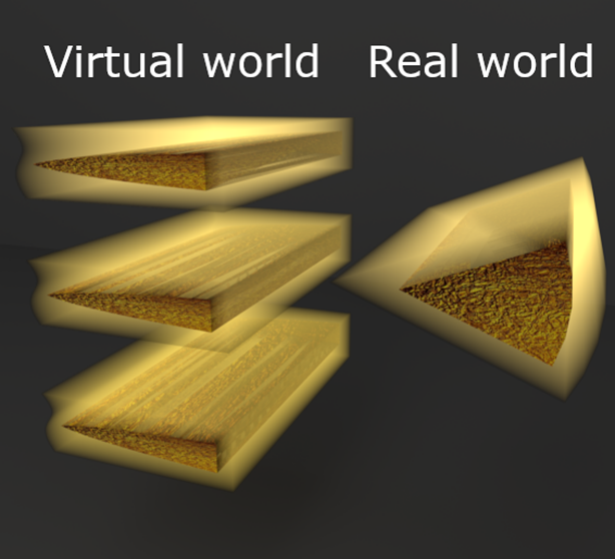

Current multiscale plasmonic systems pose a modeling challenge. Classical macroscopic theories fail to capture quantum effects in such systems, whereas quantum electrodynamics is impractical given the total size of the experimentally relevant systems, as the number of interactions is too large to be addressed one by one. To tackle the challenge, in this paper we propose to use the Madelung form of the hydrodynamic Drude model, in which the quantum effect electron spill-out is incorporated by describing the metal–dielectric interface using a super-Gaussian function. The results for a two-dimensional nanoplasmonic wedge are correlated to those from nonlocal full-wave numerical calculations based on a linearized hydrodynamic Drude model commonly employed in the literature, showing good qualitative agreement. Additionally, a conformal transformation perspective is provided to explain qualitatively the findings. The methodology described here may be applied to understand, both numerically and theoretically, the modular inclusions of additional quantum effects, such as electron spill-out and nonlocality, that cannot be incorporated seamlessly by using other approaches.

## Introduction

The recent unprecedented advancements in nanofabrication have allowed both the scientific and industrial communities to push the boundary of nanoscale systems^1−4^ and to utilize one of the most impressive features of plasmonics: the ability to go beyond the diffraction limit.^5^ Hence, plasmonics is now a buoyant field in physics, engineering, and chemistry, both fundamental^6−8^ and applied research^9−16^ alike. However, with these advances, a problem appears; the classical models for plasmonics can, sometimes, become insufficient to describe multiscale system involving micro and nano scales in which electron confinement approaches a length scale of the order of the Fermi wavelength of the valence electrons.^17,18^ Similarly, quantum electrodynamics are still prohibitive for such mesoscopic systems due to the amount of interactions they need to account for as a full quantum model would need to describe each electron in the system as well as their interactions, leading to a complex many-body problem.^19^

The classical macroscopic theory for plasmonics uses the Lorentz–Drude model to describe the metal and Maxwell’s equations to describe the electromagnetic field.^20^ It still satisfyingly describes the majority of the plasmonic systems today. However, the moment that plasmons show some quantum features (for example, when a relevant scale of the system is of the order of the nanometer) then the classical model falls apart.^21^ To solve this problem, several methods have been proposed over the years, ranging from the most widely adopted hydrodynamic Drude model (HDM)^22,23^ to those that spring from density functional theory such as the density functional tight binding.^24,25^ The HDM is a semiclassical model that describes the electrons in a metal under the influence of a electromagnetic field as a fluid; it can describe some quantum phenomena while still holding some of the simplicity of the classical models.^26,27^ By describing the electrons in a metal with the fluid equations, the HDM can take into account atomic and subatomic interactions and has also the ability to be nonlocal—one of the most integral problems in modern day plasmonics.^28−30^ A model is nonlocal when the description of a system does not take into account the interactions of a very finite local but takes into account the interactions of the whole system.^18^ In plasmonics, this means that instead of taking into account only one electron at a time, a nonlocal plasmonic model takes into account the interactions of all the electrons of the full system.^28−30^

Inspired by the importance of plasmonic devices and the need of including nonlocal effects in plasmonic systems, in this paper we go a step further into the HDM and use it in association with the Madelung formalism, following the path set up in parallel in refs (31) and (32). Initially, the Madelung formalism was used to convert the Schrödinger equation into the fluid equations. These equations would describe the flow of probability of the wave function in quantum systems.^33^ However, our objective is to use the Madelung formalism on the fluid equations to describe them as a nonlinear Schrödinger equation. This enables us to apply quantum optics methods in our system and to have a quantum HDM in a form appropriate to numerical simulations. Even though this approach transforms the fluid equations into a nonlinear Schrödinger equation, where both approaches are hard to solve analytically, our methodology has numerous advantages. First, unlike the standard approach to HDM,^22,23,27,34^ we do not have to define additional boundary conditions to describe the geometry of our system. In our approach, this is done straightforwardly with the linear potentials in the Schrödinger equation.^35^ In this paper, to capture electron spill-out^29,36−38^ in the model, the interface between the metal and dielectric is described by a super-Gaussian function; a similar approach was followed in refs (39) and (40), but for an electron density profile following a linear polynomial and a tanh^2^ form, respectively. Note that those approaches coupling the hydrodynamic transport equations and Maxwell’s field equations that avoid additional boundary conditions do it at the expense of computational complexity.^39,41−43^ Second, using our approach, we have a Hamiltonian description of our system, which means that we can solve it modularly. Thus, we can solve first for the linear coefficients of the Hamiltonian and only after introducing the nonlinear terms.

In the following sections, we provide a linear analysis of the Madelung HDM of a nanoplasmonic wedge (translationally invariant along the out-of-plane direction) that incorporates spill-out and compare an analogous of the Fermi’s golden rule with the absorption cross section obtained by nonlocal full-wave numerical calculations. To give a physical intuition into the problem, we also provide a description of the system using conformal transformation.^28,44−47^ The wedge geometry is chosen to illustrate the work because of two reasons: (1) it is widely used experimentally due to its strong field enhancement at the apex, and (2) it can straightforwardly be treated within the conformal transformation frame.

## Theory and Computational Details

### Hydrodynamic Drude Model and the Madelung Formalism

The HDM assumes that the electrons of the metal under the influence of an electromagnetic field can be described by using the fluid equations.^48^
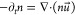
1
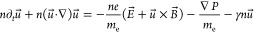
2where *n*, *u⃗*, *m*_e_, and *e* are the electron density, velocity, mass, and charge, respectively, and *E⃗* and *B⃗* are the electric and magnetic fields of the electromagnetic radiation. The terms on the right-hand side of eq 2 represent the Lorentz force, the Thomas–Fermi pressure (a pressure that accounts for the Pauli exclusion principle, with , where ℏ is the reduced Planck’s constant), and the damping forces (a phenomenological parameter that accounts for the damping due to electron–ion collisions), respectively.

By applying the Madelung formalism, one can transform eqs 1 and 2 into a nonlinear Schrödinger equation that takes the form^31^
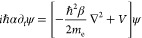
3where α and β are normalizing constants to operate with arbitrary units (a.u.) and ψ is the wave function that takes the form of , where  can be traced back to the velocity |*u⃗*|;^31^ the potential *V* is given by
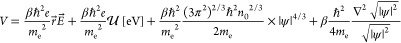
4where , and it is found after comparing eq 1 with the real part of eq 3 and eq 2 with the imaginary part of eq 3 under the assumption that the magnetic component of an electromagnetic wave can be neglected such that . For a treatment without this latter approximation, the reader is referred to the recent work ref (32).

**Figure 1:**
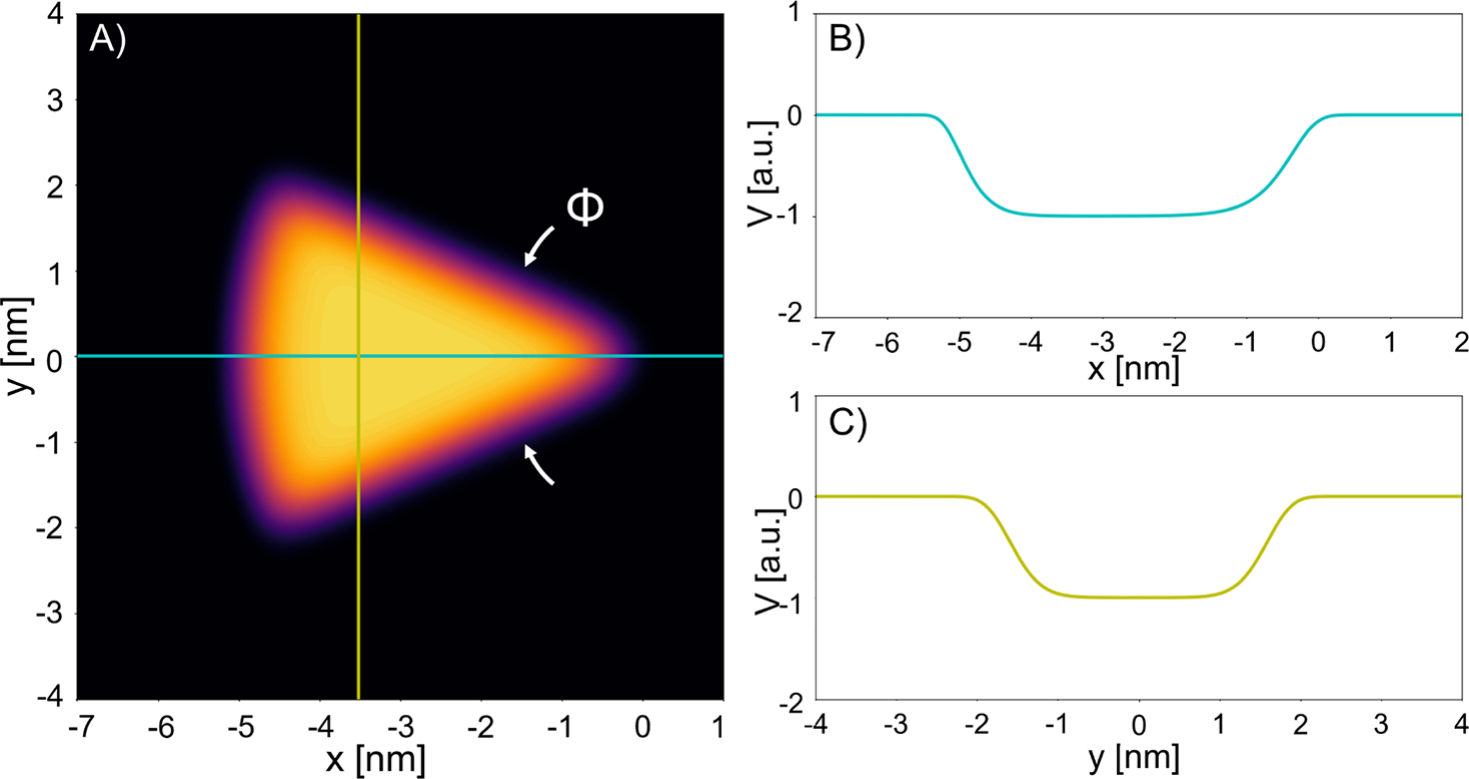
(A) Representation of the potential used to describe the two-dimensional, 2D, nonlocal nanoplasmonic wedge. Representation of the potential at *y* = 0 section (B) shown in cyan in panel A and at *x* = −3.5 section (C) shown in yellow in panel A.

Equation 4 shows the full potential needed to completely describe a plasmonic system by using the HDM with a Schrödinger equation. The first two terms are linear: the first one describes the interaction of the electric field with our system (to do this transformation, we assumed that the magnetic field is negligible), while the second one determines the geometry used. Using the function , we define the geometry of our system as a potential well.^35^ Here, we choose the geometry to be a circular sector, also termed wedge, whose boundary can be described with super-Gaussian functions (Figure 1A) to model heuristically the electron spill-out.

The representation of the potential defined with the super-Gaussian is shown in Figure 1. This super-Gaussian is mathematically defined for the arc of the wedge as
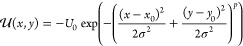
5where σ = 1, and *p*, the sharpness of the super-Gaussian function, is arbitrarily set to 10 to have a high roll-off akin to the literature.^36,49,50^*U*_0_ is the value of the maximum of the potential (*U*_0_ = 1 in Figure 1, but for the numerical calculations *U*_0_ = 25000 a.u. to ensure convergence of the first seven bound energy states; see the next subsection for further discussion on the choice of *U*_0_), and *x*_0_ = 0 and *y*_0_ = 0 define its centers with a radius of 5 nm. For the radii of the wedge, the same super-Gaussian profile is defined, but normal to the radii.

The potential at *y* = 0 (that is, along the horizontal cyan line on the left-hand side of Figure 1) is shown on the right-hand side of Figure 1 to show how the super-Gaussian is drawn. As it is observed, the profile follows a steplike function. However, note that the edges are not sharp as the super-Gaussian function makes the boundary between metal and background (assumed to be air for our COMSOL Multiphysics simulations; technical details of the simulations can be found in the last subsection of Theory and Computational Details) smooth. By doing this, we can mimic a more realistic structure as at the nanoscale, the boundary of the metal system is not well-defined and allows for the electrons to “leave” the metallic system and then be pushed back.

With the potential well-defined, we can now solve the Schrödinger equation using an in-house finite difference method approach coded in Python through diagonalization with 2^10^ points along with sparse matrices to reduce memory requirements. In doing so, we push the analogy between plasmonic system and quantum mechanics and can now apply known methods of quantum optics to our system. To obtain a proxy for the macroscopic absorption cross-section property based on Fermi’s golden rule as described below, we linearized eq 4 as a first-order approximation. It is also common to organize the Hamiltonian into two parts.^51,52^ The first part (*H*_0_) is composed of the kinetic terms and the potential defined by eq 5. The second part (*H*_I_) is composed by the interaction Hamiltonian, which in our case is the dipole interaction.

To have a proxy for the absorption cross section, we use Fermi’s golden rule in the rotating wave approximation which states that
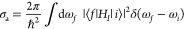
6where |*i*⟩ and |*f*⟩ are solutions to the Schrödinger equation if the Hamiltonian was only defined by *H*_0_. One should note that the presence of the δ(ω_*f*_ – ω_*i*_) represents the density of states at the energy *E*_*f*_. Here, we are assuming that this is 1. Hence, we are considering that the power transferred from the incident light source to the plasmonic system is constant and total. A refinement of this value is beyond the scope of this paper, and it will be addressed elsewhere.

**Figure 2:**
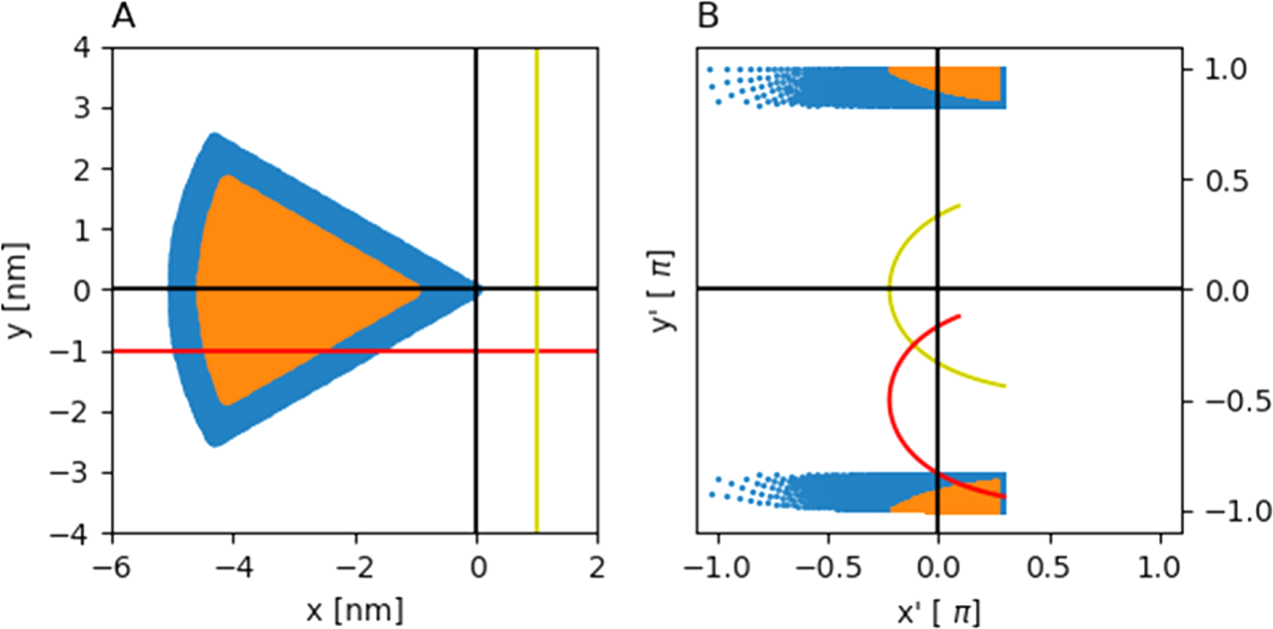
Representation of the 2D geometry used in COMSOL Multiphysics and its transformation into the virtual world following the conformal transformation *z*′ = ln(*z*), with *z* = *x* + *iy* and *z*′ = *x*′ + *iy*′. The yellow and red lines are visual aids of the transformation.

Our Madelung results are compared to COMSOL Multiphysics simulations, whose basic implementation is illustrated by Figure 2A: a circular sector translationally invariant along the out-of-plane direction, with radius of 5 nm, and with the central angle ϕ from 0.4 to 1.1 rad; we restrict ourselves to this central angle range as it is the one in which convergence is achieved for the Madelung HDM, as discussed later on. The illumination is a plane-wave propagating along *y* and polarized along *x*. In this numerical study, we use a popular COMSOL Multiphysics implementation of the HDM introduced by Toscano et al.^53^ This implementation divides the geometry in two section. The blue shell in Figure 2A defines the nonlocal domain sized to match the transition of the super-Gaussians in our Madelung-based theory; the orange one is defined by the bulk properties of the metal.

To provide a complementary view on the problem that will help with the interpretation of the system’s response, we resort into conformal transformation. Applying the conformal transformation *z*′ = ln(*z*) to our wedge system, whereby circles and radial lines in the *z*-plane are converted to vertical and horizontal lines in the *z*′-plane, respectively,^10,46^ leads to Figure 2B. As one can see, the transformation extends the local and nonlocal domains nonuniformly in the virtual world. Although this prevents us to solve the problem analytically, the virtual space, which retains the electromagnetic properties of the real world by virtue of the Maxwell’s equations invariance under arbitrary coordinate transformations, reveals that the solution of this nonlocal system will departure significantly from the local one as the virtual world is no longer a periodic insulator/metal/insulator heterostructure (translationally invariant along the out-of-plane direction). Further discussion is given in the Results and Discussion.

### Choice of Potential *U*_0_

**Figure 3:**
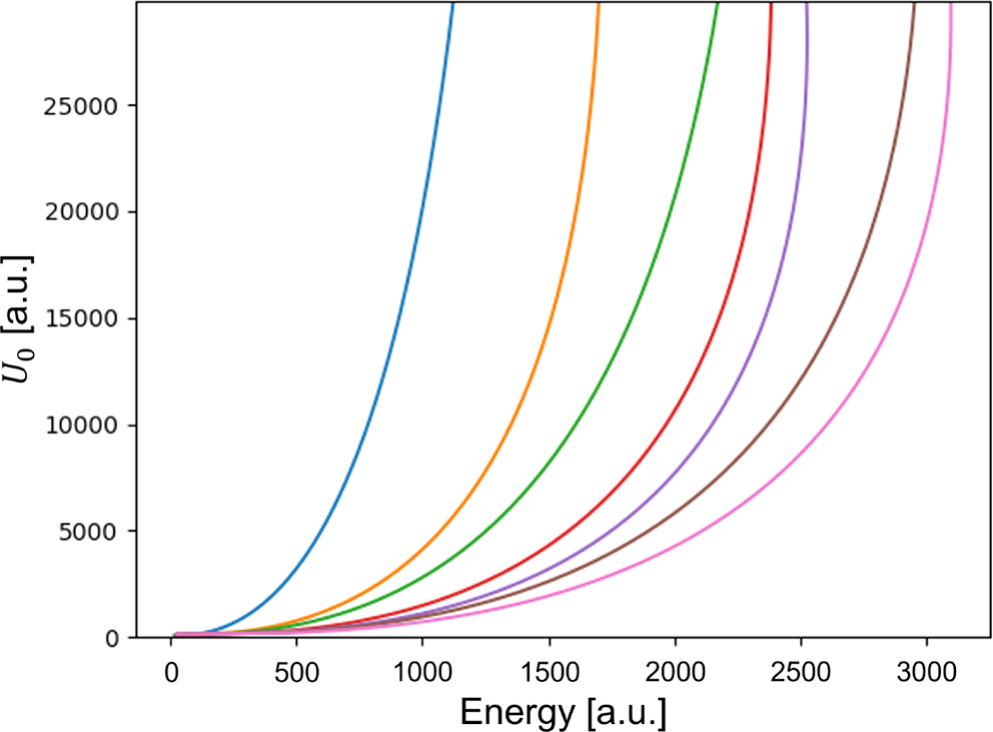
Energy values of the system against the choice of maximum potential for the first seven levels.

Figure 3 shows the energy values of the system against the choice of maximum potential. The seven energy levels reach their asymptotic regime for *U*_0_ > 20000. Hence, this paper considers *U*_0_ = 25000 for its calculations. *U*_0_ is ultimately related to the Wigner–Seitz parameter which defines the type of metal. However, finding such a connection through the Jellium model is beyond the scope of this work and is left for the future.

### COMSOL Multiphysics Simulations

Simulation results are calculated by using the commercial finite element analysis software COMSOL Multiphysics. The model of metal follows an ideal metal whose concentration of free electrons per unit of volume is 1.07 × 10^28^ and a dielectric permittivity of
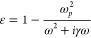
7where ω_*p*_ = 5.83563 × 10^15^ rad/s and γ = 2.861364884 × 10^14^ rad/s.^31^

After convergence tests, the simulation box of both local and nonlocal full-wave simulations is a cylinder of radius *r* = 150 nm. Scattering boundary conditions and perfectly matched layers were applied around this box to avoid reflections. A refined mesh with a minimum length of 0.2 and 0.1 nm is used to ensure accurate results for the local and nonlocal simulations, respectively. The excitation is a plane-wave propagating along *y* and polarized along *x*.

## Results and Discussion

**Figure 4:**
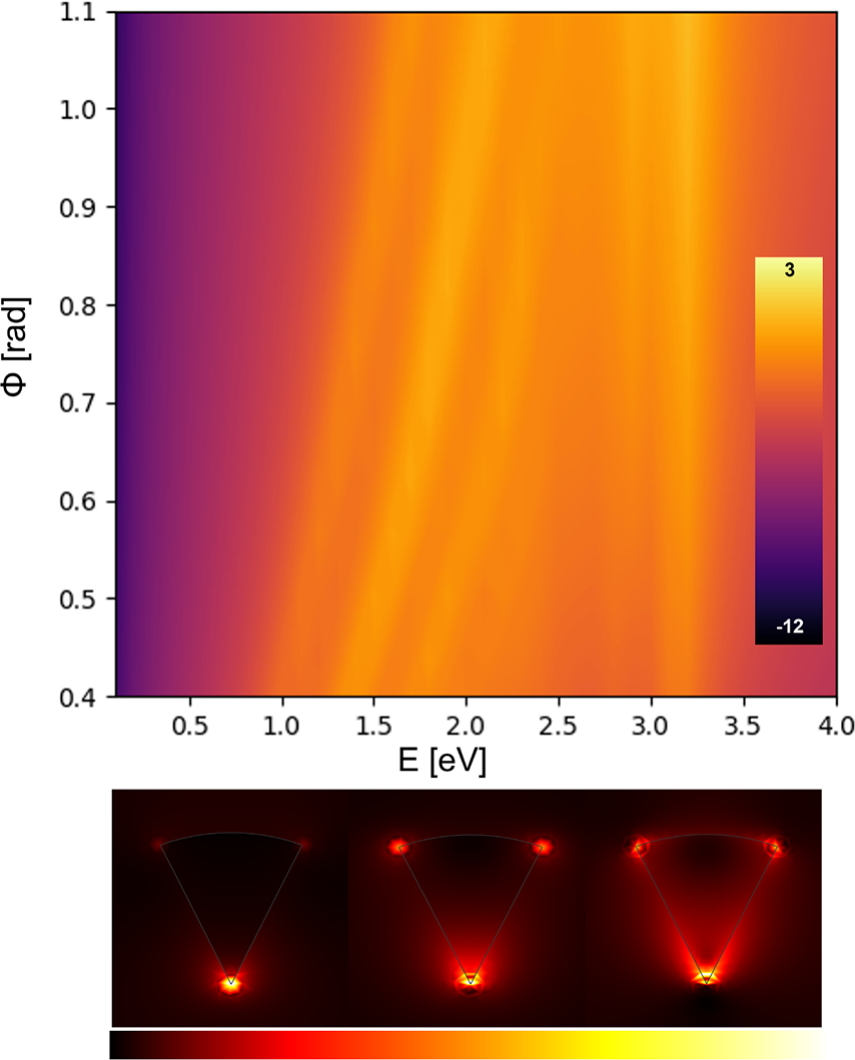
(top) Local absorption cross section in log scale. (bottom) Intensity distribution for the fundamental (left), second (middle), and third (right) mode for ϕ = 0.8 rad, whose corresponding energies are 1.44, 1.86, and 2.26 eV, respectively.

To have a reference point and facilitate subsequent discussion, we compute the absorption cross section for the classical electromagnetic solution and plot the intensity distribution for the first three localized plasmon modes (see Figure 4). All these localized plasmon modes blue-shift as a function of ϕ. This is consistent with the scenario described by the virtual world (i.e., periodic insulator/metal/insulator heterostructure) (see Figure 2B) yet consider the slabs to be homogeneously orange, wherein thicker metal slabs (i.e., wider ϕ) result in a weaker coupling between the so-called long-range and short-range plasmons and, thus, higher energy (i.e., blue shift) to the fundamental short-range plasmon.^54^

**Figure 5:**
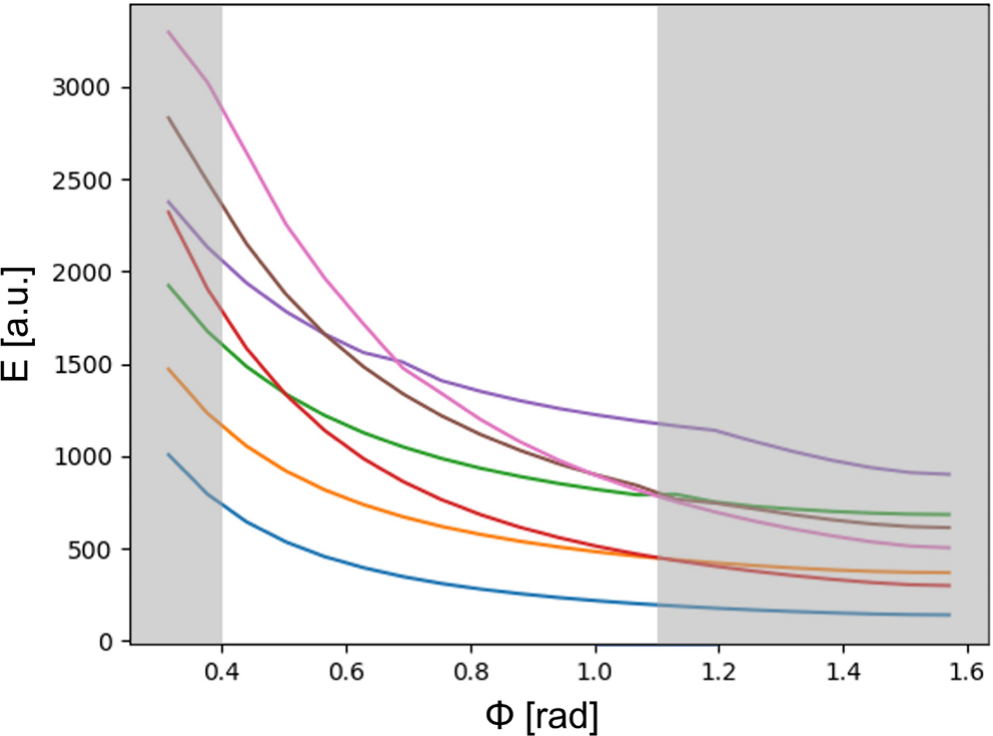
First seven energy levels of the geometry defined as a function of the circular sector central angle ϕ, where each color represents a different state. The clear zone represents the truncated parameter sweep where convergence is ensured.

With the local scenario described as a benchmark, we can now move to the nonlocal scenario. To this end, we first solve the linearized Schrödinger equation and identify the range of ϕ where our approach converges. Figure 5 shows the eigenvalues of eq 3, which are analogous to the energy values of each state, as a function of the central angle of the circular sector ϕ. The main differences between these eigenvalues and the energy levels of a potential well are the crossover of some states. If we consider that the order of each state is defined as it is when ϕ < 0.4 rad, then the fourth state (represented in red) crosses over with the third one (represented in green) at ϕ = 0.5 rad. The fifth (represented in purple), sixth (represented in brown), and seventh (represented in pink) states also show several crossing among themselves and with the third state (green) within the expanded ϕ range displayed in Figure 5. However, in the range 0.4 ≤ ϕ ≤ 1.1 rad, all crossings happen among the seven energy levels, which is a necessary condition to avoid nonphysical artifacts in the context of eq 6. Notice that for ϕ ≈ 0.39 rad there is an incomplete crossing in the seven energy level (pink), whereas for ϕ ≈ 1.2 rad there is another incomplete crossing for the fifth energy level (purple). Thus, in such cases, eq 3 would not be considering the correct first number of states. Hence, we gray ϕ < 0.4 rad and ϕ > 1.1 rad and ignore such a range for the following results and discussion. It is also worth pointing out that the small inflection of the fifth energy level (purple) at ϕ ≈ 0.7 rad is due to unavoidable computational error and not an actual crossing with a different energy level.

**Figure 6:**
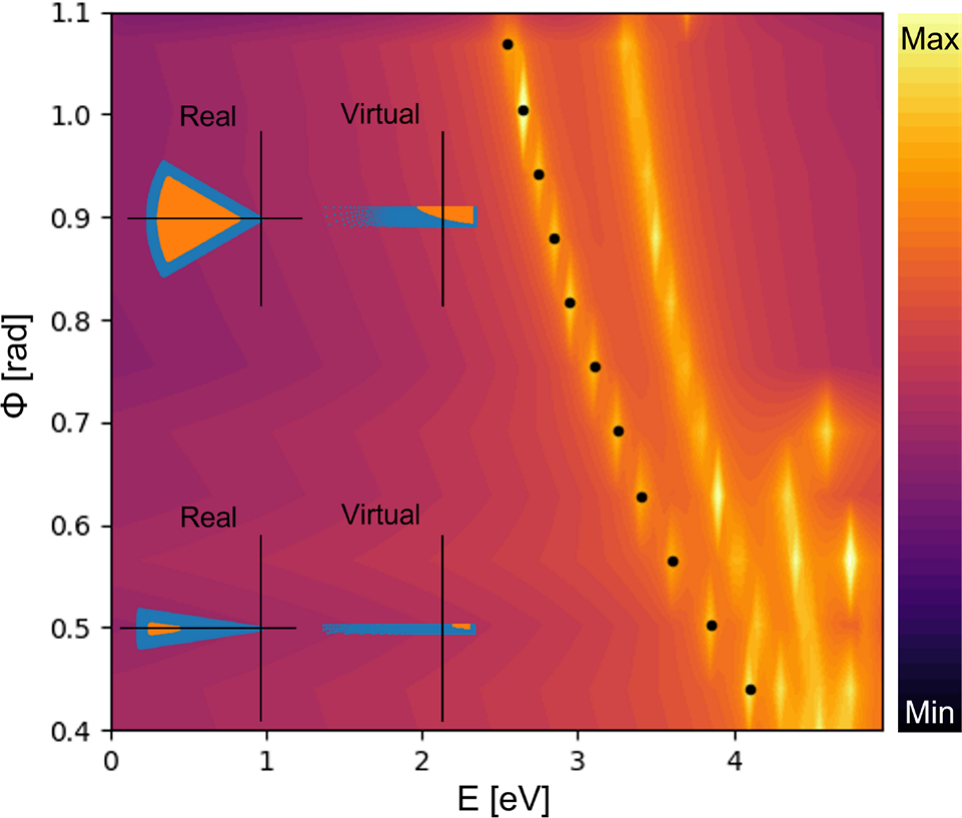
Proxy for absorption cross section σ_a_ in log scale, calculated by using the first seven energy levels in eq 6. The dots represent the maximum value of the absorption. The energy values on the *x*-axis are the energy of light considered.

After solving the Schrödinger equation, one has everything needed to solve eq 6. Figure 6 represents the absorption cross section defined by eq 6. One should note that the energy values encountered in the *x*-axis come from the interaction Hamiltonian *H*_*I*_, making it possible for us to find the resonances of the system. The two insets show the geometry with two different angles along with the corresponding virtual world. The dots represent the maximum of the absorption cross section which correlates to the first plasmonic mode of the system. By increasing the angle ϕ, the energy of the resonance decreases, which could be found counterintuitive from our preliminary local analysis (Figure 4), previous local studies of bowties translationally invariant along the out-of-plane direction (i.e., two-dimensional bowties),^44,47^ and even three-dimensional bowties under out-of-plane illumination;^55^ this will be discussed later in the text. At low values of ϕ this resonance is closer to the plasmonic frequency which increases the interactions of different modes. To account for this interaction, following our approach, one would need to increase the number of energy levels calculated. However, because of computational restrictions, we have only used the first seven levels. This stronger mode interaction is clearly shown in Figure 6 for values of ϕ lower than 0.5 where it is difficult to distinguish between the first mode (marked by the black dots) and the next one (unmarked).

**Figure 7:**
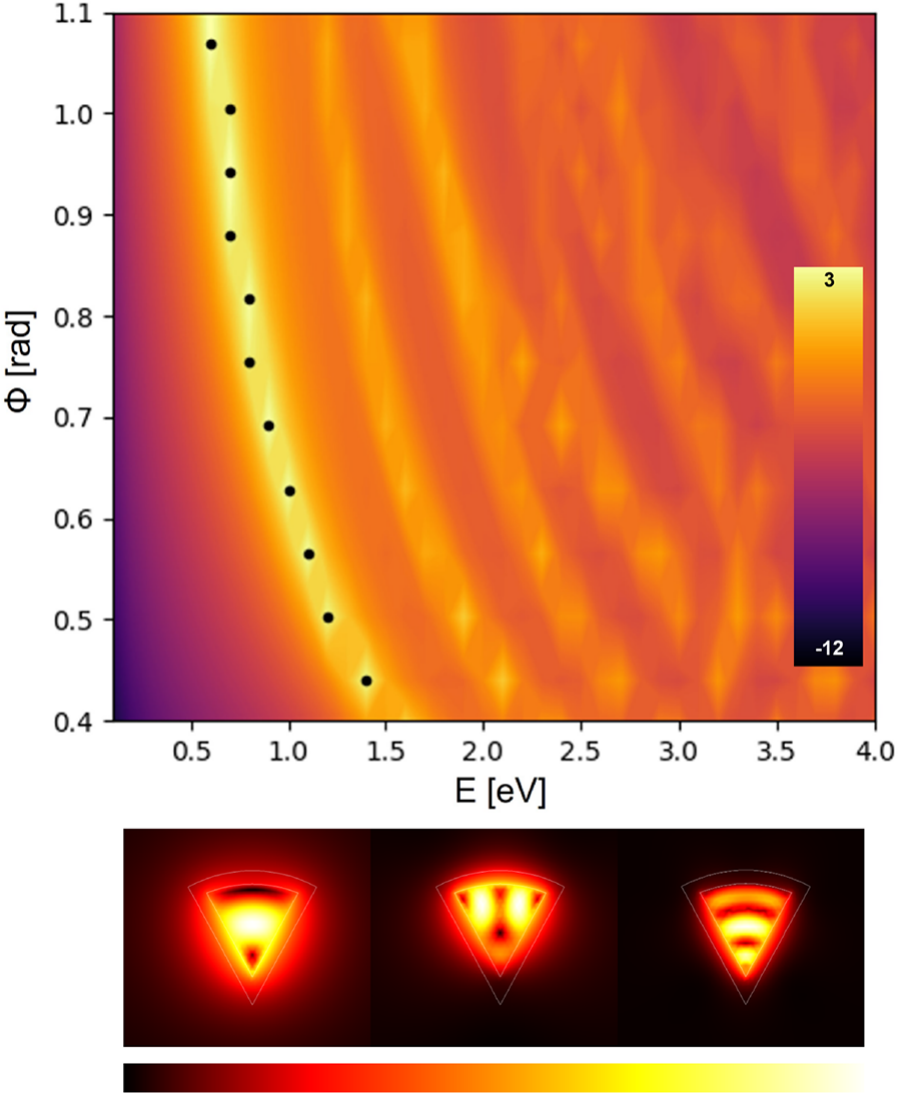
(top) Nonlocal absorption cross section in log scale calculated using the popular hydrodynamic model finite-element implementation by Toscano et al.^53^ (bottom) Intensity distribution for the fundamental (left), second (middle), and third (right) mode for ϕ = 0.8 rad, whose corresponding energies are 0.79, 1.39, and 1.91 eV, respectively.

To test these results, we solve the same geometry using the popular COMSOL Multiphysics implementation of the HDM^53^ and assume it as the ground truth. Figure 7 shows the values of the absorption cross section in log scale. The dotted values represent again the maximum value of the absorption cross section that also coincide with the first plasmonic mode of the system. It is worth mentioning as well that for the higher values of the angle ϕ some consecutive dots appear to be aligned in a vertical line. This is because of a computational error due to our computational resources, as to improve this one would need to use a finer mesh. For completeness, the intensity distribution of the first three fundamental modes for ϕ = 0.8 rad is depicted at the bottom of Figure 7.

Comparing both Figures 6 and 7, one can see that, aside from a shift in energy, both models predict that the response of our plasmonic system takes the same form, especially for the first plasmonic mode, with a red shift with increasing angle ϕ unlike the local model (Figure 4). The conformal transformation perspective provides an elegant explanation for this as insinuated earlier. In local models, increasing the angle ϕ will blue-shift the energy as can be seen in refs (44, 47, and 55). That trend can be rooted in the dispersion relation of the coupled odd and even surface plasmon modes for insulator/metal/insulator heterostructures:^54^ the thinner the metal layer is, the lower the energy of the even mode is. It is precisely such heterostructure (yet periodic) that appears when applying the conformal transformation *z*′ = ln(*z*). In the nonlocal case, however, we see a red shift because of the impact of the nonlocality modeled by the dielectric layer (i.e., the blue shell) mapping the surface charge smearing. In the leftmost insets of Figure 6 one can see that there is a nonlinear reduction of the proportion of the bulk local metal (orange area) compared to the nonlocal part (blue area) as the angle ϕ decreases. This nonlinear relationship between angle ϕ and bulk local metal is even further magnified when one compares the virtual worlds (see the right-hand side of the insets in the same Figure 6). Indeed, now one should not treat the virtual world as a periodic insulator/metal/insulator heterostructure as in full local models but as a nanoparticle (array) embedded in a nonhomogeneous dielectric environment. In such a situation, the fundamental plasmon resonance is governed by the localized plasmon of the nanoparticle and red-shifts with nanoparticle size (i.e., with angle ϕ for our triangular nanowire). Consequently, from all the above discussions, the intensity distribution of the fundamental modes is dramatically different between the local (Figure 4) and nonlocal model (Figure 7).

The comparison of Figures 6 and 7 also highlights the advantages and disadvantages of both our Madelung HDM and the hydrodynamic model in finite-element simulation implementation. On one hand, Figure 6 shows the simplicity of the model that predicts the outcome at the cost of the higher order modes. This is due to the limiting of the number of energy levels calculated. On the other, the model of Figure 7 can calculate higher order modes and their interaction at the cost of computational power and a more intricate, and by extension more difficult to solve, system of equations. This is shown by the previously mentioned computational error that vertically aligned some of the maxima.

**Figure 8:**
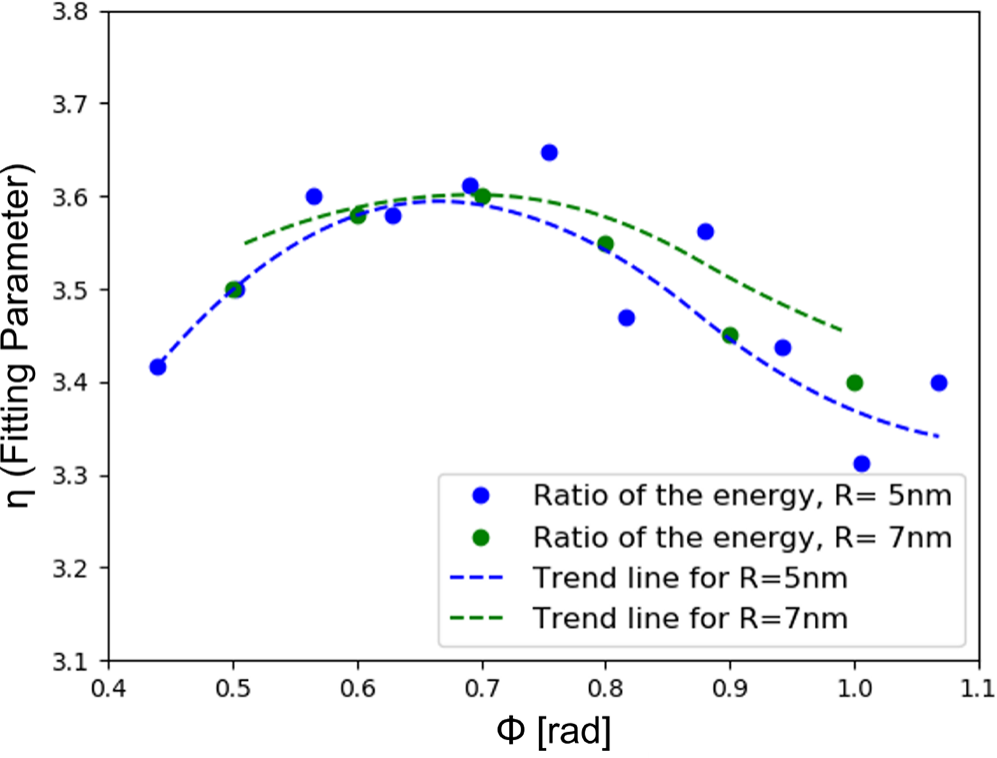
Comparison between COMSOL Multiphysics model and HDM with the Madelung formalism for 5 nm (blue) and 7 nm (green) radius circular sectors. The dots are the ratio of the energy of the absorption cross-section maximum obtained from each model, and the lines are the trends.

Although the hydrodynamic model excludes consideration of specific quantum phenomena, including tunneling and quantum oscillations, it captures much of the microscopic dynamics in nanoplasmonics and provides predictions in agreement with experiments. Hence, we assume its COMSOL Multiphysics implementation to be ground truth and compute a correction factor η to reconcile the resonance energy between our theory and the hydrodynamic model in finite-element simulation. Figure 8 shows that η is more stable when compare to our previous work,^31^ as its absolute value fluctuates over a smaller interval raging from 3.30 to 3.65. This is to be expected with the application of super-Gaussians to define the geometry. With these types of potentials the model is nonlocal, as they introduce a dependency on position to the potential instead of an on/off type of approach and a spill-out effect as well. The fact that this ratio is not constant at the value 1 means that the nonlinear terms in the potential that were not considered still retain some nonlocality present in the original model from Toscano et al.,^53^ however small.

To stress test the model presented here, the same analysis is done for a 7 nm radius circular sector. As one can see in Figure 8, aside from computational errors, both models react to the change in a very similar way, thus proving that once this correction factor η is found for one geometry, it can be used for other sizes.

## Conclusion

In this article, the electron spill-out has been incorporated in a linearized quantum hydrodynamic model encapsulated on a single Schrödinger equation by defining a potential following a super-Gaussian function at the metal–dielectric interfaces. The quantum hydrodynamic model was obtained by rewriting the set of continuity and Euler equations using Madelung transformation. The model was specifically used to look at the electrodynamics of a triangular nanowire, and the results were correlated to a popular linearized hydrodynamic Drude model implemented by a finite-element method. The discussion was supplemented with a conformal transformation perspective to provide an elegant qualitative physical understanding on the findings and justify the opposite electromagnetic response observed between classical local and nonlocal models against the central angle of the circular sector. Although the scenario analyzed here is two-dimensional, and thus does not capture spill-out in the out-of-plane direction that may be relevant in experiments, the core of the work is a finite difference method that can be made easily three-dimensional; this extension is left for future work.
